# Comparison of operative outcomes between monopolar and bipolar coagulation in hepatectomy: a propensity score-matched analysis in a single center

**DOI:** 10.1186/s12876-022-02231-y

**Published:** 2022-03-29

**Authors:** Ryuta Muraki, Yoshifumi Morita, Shinya Ida, Ryo Kitajima, Satoru Furuhashi, Makoto Takeda, Hirotoshi Kikuchi, Yoshihiro Hiramatsu, Atsuko Fukazawa, Takanori Sakaguchi, Mayu Fukushima, Eisaku Okada, Hiroya Takeuchi

**Affiliations:** 1 Department of Surgery, Hamamatsu University School of Medicine, 1-20-1 Handayama, Higashi-ku, Hamamatsu, Shizuoka 431-3192 Japan; 2 Department of Perioperative Functioning Care and Support, Hamamatsu University School of Medicine, 1-20-1 Handayama, Higashi-ku, Hamamatsu, Shizuoka 431-3192 Japan; 3 Department of Gastroenterological Surgery, Iwata City Hospital, 512-3 Ohkubo, Iwata, Shizuoka 438-8550 Japan; 4 Department of Diagnostic Pathology, Hamamatsu University School of Medicine, 1-20-1 Handayama, Higashi-ku, Hamamatsu, Shizuoka 431-3192 Japan; 5 Faculty of Social Policy and Administration, Hosei University, Aihara 4342, Machida, Tokyo 194-0298 Japan

**Keywords:** Hemostatic device, Hepatectomy, Intra-abdominal infection, Ascites, Propensity score matching

## Abstract

**Background:**

Various hemostatic devices have been utilized to reduce blood loss during hepatectomy. Nonetheless, a comparison between monopolar and bipolar coagulation, particularly their usefulness or inferiority, has been poorly documented. The aim of this study is to reveal the characteristics of these hemostatic devices.

**Methods:**

A total of 264 patients who underwent open hepatectomy at our institution from January 2009 to December 2018 were included. Monopolar and bipolar hemostatic devices were used in 160 (monopolar group) and 104 (bipolar group) cases, respectively. Operative outcomes and thermal damage to the resected specimens were compared between these groups using propensity score matching according to background factors. Multivariate logistic regression analysis was performed to identify predictive factors for postoperative complications.

**Results:**

After propensity score matching, 73 patients per group were enrolled. The monopolar group had significantly lower total operative time (239 vs. 275 min; *P* = 0.013) and intraoperative blood loss (487 vs. 790 mL; *P* < 0.001). However, the incidence rates of ascites (27.4% vs. 8.2%; *P* = 0.002) and grade ≥ 3 intra-abdominal infection (12.3% vs. 2.7%; *P* = 0.028) were significantly higher in the monopolar group. Thermal damage to the resected specimens was significantly longer in the monopolar group (4.6 vs. 1.2 mm; *P* < 0.001). Use of monopolar hemostatic device was an independent risk factor for ascites (odds ratio, 5.626, 95% confidence interval 1.881–16.827; *P* = 0.002) and severe intra-abdominal infection (odds ratio, 5.905, 95% confidence interval 1.096–31.825; *P* = 0.039).

**Conclusions:**

Although monopolar devices have an excellent hemostatic ability, they might damage the remnant liver. The use of monopolar devices can be one of the factors that increase the frequency of complications.

**Supplementary Information:**

The online version contains supplementary material available at 10.1186/s12876-022-02231-y.

## Background

Blood loss and transfusion during hepatic surgery increase morbidity and mortality [1]. Severe postoperative complications worsen long-term prognosis in patients with hepatic malignancy. Several surgical methods for hepatic transection and coagulation are currently available to minimize intraoperative blood loss [2, 3]. Classical resection techniques include finger fracture, sharp dissection, and crush clamping [4, 5]. Recently, an ultrasonic surgical aspirator has been used to provide a rapid and safe operative procedure [6]. Furthermore, various hemostatic coagulation and cutting devices, such as ultrasonic scalpels, have been utilized for the transection of the liver parenchyma, and advancements in these devices have been made over the past decades [7–10]. Owing to these techniques and perioperative care, the postoperative mortality rate was reduced to 3.7%. However, the morbidity rate (25.7%) remains unsatisfactory [11].

A soft coagulation system with a monopolar electrode is a novel hemostatic device that delivers a computer-controlled low voltage without electrical discharge; heat is transferred to the deeper areas of the liver while preventing the carbonization of tissues [12, 13]. However, heat injury to the remnant liver caused by this device remains a concern. Deep thermal damage may lead to liver necrosis and bile leakage postoperatively, which can result in morbidity [14].

A saline-coupled bipolar forceps coagulation system is also considered to be a safe and reliable hemostatic device to decrease intraoperative hemorrhage [15]. It has also been widely used by neurosurgeons, as it can safely cauterize small blood vessels adjacent to a nerve without causing damage if the operator does not pinch the neural tissue [16, 17]. By contrast, the coagulative effect of bipolar coagulation is weaker than that of monopolar coagulation and tends to prolong the transection time [18].

Although some studies have investigated the role of hemostatic devices in hepatic transection, a comparison between monopolar and bipolar coagulation, particularly their usefulness or inferiority, has been poorly documented [3, 15, 18]. The purpose of this retrospective cohort study was to investigate whether the monopolar device has better hemostatic efficiency than the bipolar device and whether the monopolar device increases postoperative complications.

## Materials and methods

### Patients

**Fig. 1:**
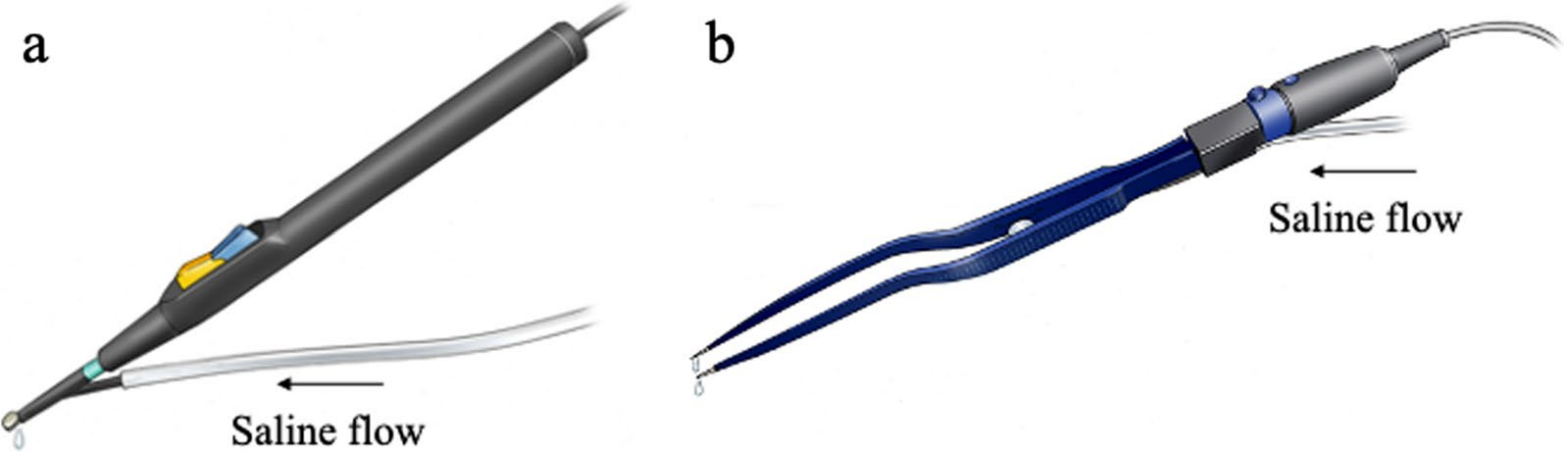
Hemostatic devices. During coagulation of the liver parenchyma, saline is dripped through the electrode tip to prevent the adherence of clots. The end of the tube is connected to a saline bottle for drip infusion. **a** monopolar device, **b** bipolar device

From January 2009 to December 2018, 337 consecutive patients underwent hepatic resection at Hamamatsu University School of Medicine, Japan. Eligibility criteria included a scheduled open liver resection for benign or malignant hepatobiliary disease requiring a transection of the liver parenchyma with the Cavitron Ultrasonic Surgical Aspirator (CUSA; Valleylab, Boulder, CO, USA). The hemostatic device on the cutting liver surface used saline-coupled soft coagulation of the IO advanced monopolar electrode with the VIO 300 D system (Erbe Elektromedizin GmbH, Tübingen, Germany) (monopolar group) or saline-coupled bipolar forceps coagulation with the MALIS Bipolar Electrosurgical System (CMC-III, Codman; Johnson & Johnson, New Brunswick, NJ, USA) (bipolar group) (Fig. 1). Sixty-five patients who underwent laparoscopic liver resection were excluded because the monopolar electrode was used in all cases. Three patients in whom another hemostatic device was used and five who underwent surgery without the CUSA were also excluded.

All patients’ data were consecutively collected during the follow-up period. The outcomes of patients who underwent monopolar coagulation were compared with those of patients who underwent bipolar coagulation. The results were analyzed using the propensity score matching (PSM) method. Postoperative complications were classified according to the Clavien-Dindo (CD) definition [19]. Ascites was considered when the patient’s body weight or the volume of drainage fluid increased or when ultrasonography or CT revealed fluid collection. Intra-abdominal infection was suspected when the patient had severe fever or when there was an elevation of inflammation markers; this was confirmed using ultrasonography or CT scan. The definitions of ascites and intra-abdominal infection are provided in Supplementary Tables 1 and 2. Additionally, the comprehensive complication index (CCI), calculated as the sum of all complications weighted based on their severity, was evaluated [20]. Informed consent for data collection was obtained using the opt-out method on the homepage of our institution’s website (https://www.hama-med.ac.jp/research/clinical-res/erc/disclosure-info/index.html). This study was approved by the ethical review board at our institution (approval number 17–124) in accordance with the ethical guidelines for clinical studies of the Japanese Ministry of Health, Labour and Welfare.

### Surgical procedures

Hepatic transection was carried out using the CUSA under intermittent Pringle maneuver. This consisted of clamping the portal triad for 15 min in the case of a normal liver and 10 min in the case of liver dysfunction and releasing the clamp in both cases for 5-min intervals. The decision on the type of energy device used, such as Harmonic, LigaSure, and EnSeal vessel sealing systems, was made based on the surgeon’s preference. These energy devices are used for liver mobilization and lymph node dissection and have not been adapted for liver parenchymal transection. Small vessels (diameter < 2 mm) were ligated with thin (3–0) sutures or coagulated with electro cautery. Large vessels (diameter ≥ 2 mm) were ligated with thin (3–0) sutures. Glissonean branches of the primary or secondary trunk were doubly tied or ligated using a linear stapler. The isolated large hepatic vein was closed with a running suture or ligated using a linear stapler. During liver transection, bleeding from the cut surface was controlled using the hemostatic device, gentle compression, or suturing. To investigate bile leakage after liver transection, an intraoperative bile leak test was performed as previously reported [21].

### Postoperative indication for drain removal

Drain removal criteria included the following: (1) drainage volume < 200 ml, and (2) drainage fluid was not contaminated with bile juice. If the drainage volume was > 200 ml on postoperative day 7, the drain was removed when the drain fluid was aseptic. Additionally, diuretics were administered, or the drain insertion site was sutured.

### Evaluation of burned area in resected specimens

To evaluate thermal damage caused by hemostatic devices, we focused on the cutting surface of resected specimens containing the maximum tumor diameter. The specimens were fixed in 10% neutral buffered formalin. Histopathological examinations were performed in hematoxylin–eosin-stained sections. Two surgeons (SI and MT) who were blinded to any clinical information, including which hemostatic device was used, independently evaluated the burned length in resected specimens. They measured the average of three locations in formalin-fixed specimens when the edge was uniformly burned, or they measured only one location when the edge was partially burned. The average score measured by the two surgeons was adopted.

### Statistical analysis

All continuous data were expressed as mean ± standard deviation (SD) or median (range, interquartile range). The Mann–Whitney *U* test or Student’s *t*-test was used to compare continuous variables. Pearson’s chi-square test or Fisher’s exact test was used to compare categorical variables. PSM was used to correct for biases in baseline variations between the two groups. Greedy matching (1:1 ratio without replacement) using a caliper width of 0.2 SD of the logit of the estimated propensity score was performed. The propensity score was calculated based on the diagnosis, background liver disease, type of resection, lymph node dissection, biliary reconstruction, neoadjuvant chemotherapy, total bilirubin level (mg/dL) (≥ 1.1 or < 1.1), aspartate transaminase level (AST, IU/L) (≥ 31 or < 31), albumin level (g/dL) (≥ 3.9 or < 3.9), prothrombin activity (≥ 69% or < 69%), activated partial thromboplastin time (≥ 69% or < 69%), hemoglobin level (g/dL) (≥ 11.4 or < 11.4), and indocyanine green retention rate at 15 min (ICGR15) (≥ 10% or < 10%) using the logistic regression model. The threshold of the test values was based on our institution’s criteria. A linear mixed-effects model for repeated measures was used to determine the association between the hemostatic device and postoperative blood examination, including levels of AST, C-reactive protein (CRP), and albumin, on each postoperative day. Paired comparisons with Bonferroni correction were used to compare pairs of postoperative days. Significant differences between hemostatic devices were analyzed using independent-sample Student’s *t*-test or the Welch test on each postoperative day. Multivariate logistic regression analysis was performed to identify predictive factors for postoperative complications. Odds ratio (OR) and 95% confidence interval (CI) were calculated. All calculations were carried out using SPSS Statistics software version 26 (IBM Corp., Armonk, NY, USA), and *P* values < 0.05 were considered significant.

## Results

### Patients’ characteristics before PSM

**Fig. 2:**
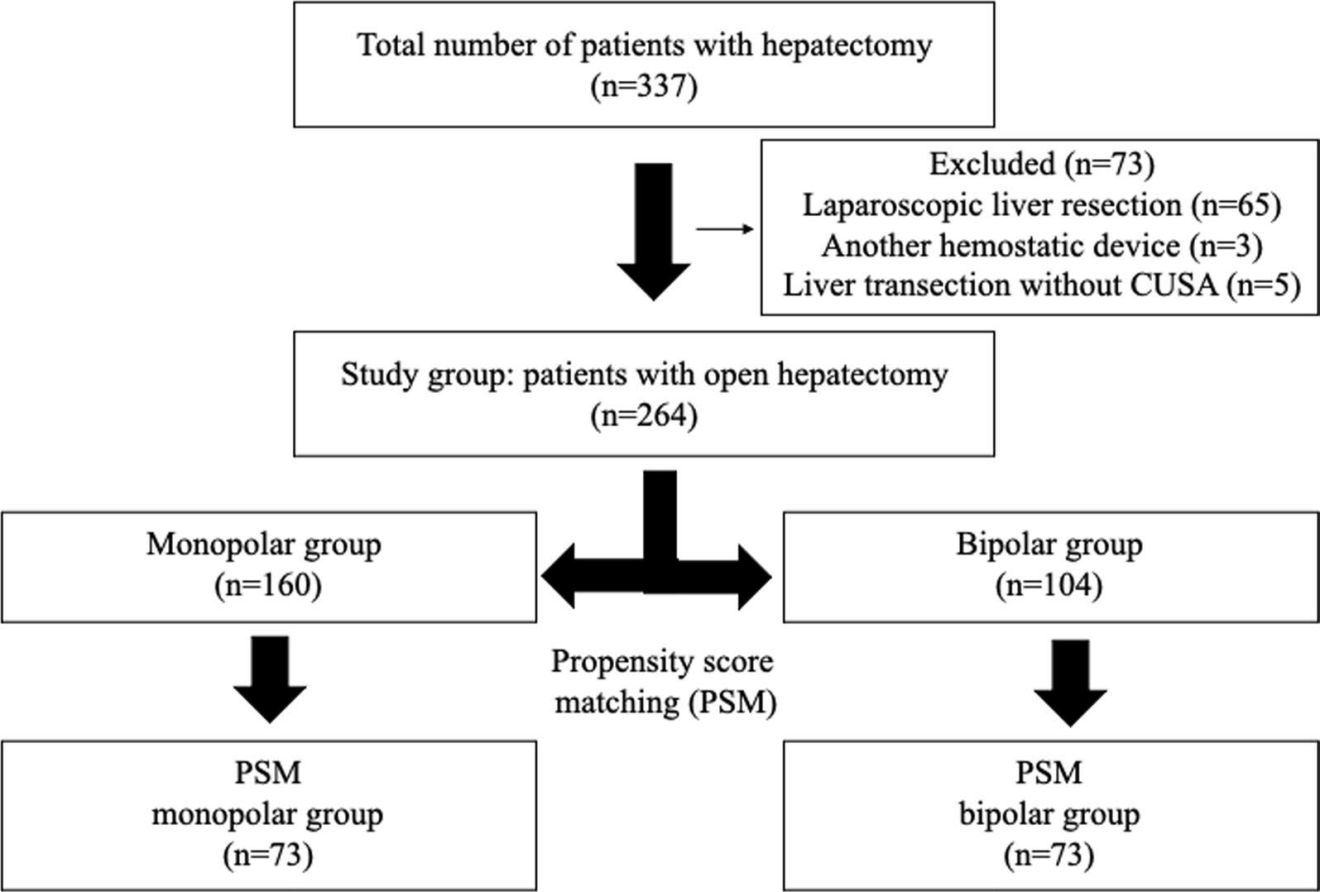
Flow diagram for the study. CUSA, Cavitron Ultrasonic Surgical Aspirator; PSM, propensity score matching

A total of 264 patients who underwent open hepatectomy were classified according to the type of hemostatic device used into the monopolar and bipolar groups (Fig. 2). Almost all patients were Japanese, except for one who was Chinese. Before November 2011, 104 patients were operated on using bipolar forceps as coagulation device. From December 2011 to December 2018, 160 patients were operated on using monopolar coagulation. The energy devices used for liver mobilization and lymph node dissection included Harmonic, LigaSure, and EnSeal vessel sealing systems; however, these were not used for liver parenchymal transection. Table 1 presents the patient background, type of surgical procedure, and laboratory data of the two groups. Some significant differences were observed between the two groups in terms of diagnosis, lymph node dissection, biliary reconstruction, AST level, albumin level, prothrombin activity, activated partial thromboplastin time, hemoglobin level, and ICGR15.

**Table 1:** Patient characteristics before propensity score matching

	Monopolar (n = 160)	Bipolar (n = 104)	*P* value
Age	68 (17–87, 14)	69 (30–85, 13)	0.745
Sex (male/female)	116/44	73/31	0.685
BMI	22.7 (12.5–32.3, 4.50)	22.7 (15.9–37.1, 4.40)	1.000
ASA (1/2/3)	14/130/16	10/79/15	0.516
Diagnosis (HCC/CCC/meta/biliary tract cancer/benign disease/other)	72/10/42/16/17/3	65/6/21/3/9/0	**0.045**
Background liver disease (normal/HCV/HBV/ASH/NASH/other)	90/43/12/4/8/3	42/43/9/3/5/2	0.135
Liver fibrosis^a^ (f0–1, f2–4)	57/56	33/51	0.120
Type of resection (partial/lateral/subsegmentectomy/segmentectomy/hemihepatectectomy/trisegmentectomy)	48/2/38/22/48/2	22/5/33/14/30/0	0.362
Number of resected lesions	1 (1–16, 0)	1 (1–3, 0)	0.970
Size of the largest nodule on preoperative imaging^b^ (cm)	2.5 (0.60–20, 3.0)	2.5 (0.50–13, 2.0)	0.776
Number of nodules on preoperative imaging^b^	1 (1–10, 1)	1 (1–10, 1)	0.868
Lymph node dissection (yes/no)	37/123	11/93	**0.010**
Biliary reconstruction (yes/no)	24/136	7/97	**0.041**
Intraoperative drain insert (yes/no)	159/1	104/0	0.606
Duration of drain placement	5 (0–223, 5)	5 (2–94, 3)	0.272
Neoadjuvant chemotherapy (yes/no/previous)	27/120/13	11/90/3	0.059
Total bilirubin (mg/dL)	0.70 (0.3–3.1, 0.30)	0.80 (0.40–1.7, 0.40)	0.285
≥ 1.1/ < 1.1	29/131	19/85	0.976
Aspartate transaminase (IU/L)	28 (13–155, 18)	35 (9–104, 26)	**0.021**
≥ 31/ < 31	67/93	62/42	**0.005**
Albumin (g/dL)	4.1 (3.0–5.0, 0.40)	3.9 (2.3–4.9, 0.50)	**0.001**
≥ 3.9/ < 3.9	122/38	58/46	** < 0.001**
Prothrombin activity (%)	100 ± 17	92 ± 15	** < 0.001**
≥ 69/ < 69	154/6	100/4	0.605
Activated partial thromboplastin time (%)	91 (24–140, 30)	81 (46–140, 24)	**0.003**
≥ 69/ < 69	131/29	84/20	0.821
Hemoglobin (g/dL)	13 ± 2.2	13 ± 1.8	**0.009**
≥ 11.4/ < 11.4	136/24	75/29	**0.011**
Platelet (× 10^4^/μL)	18.1 (3.20–61.8*,* 9.20)	16.4 (4.80–49.9, 8.60)	0.185
≥ 15.3/ < 15.3	102/58	65/39	0.837
Blood urea nitrogen (mg/dL)	14.9 (7.00–39.5, 5.60)	14.6 (7.00–27.2, 6.40)	0.423
≥ **20.0/ < 20.0**	24/136	16/88	0.932
Creatinine (mg/dL)	0.78 (0.35–5.50, 0.30)	0.75 (0.43–2.30, 0.28)	0.427
≥ **1.07/ < 1.07**	21/139	12/92	0.703
C-reactive protein^c^ (mg/dL)	0.10 (0.00–9.80, 0.18)	0.11 (0.01–24.2, 0.28)	0.439
≥ 0.14/ < 0.14	61/93	35/44	0.491
ICGR15^d^ (%)	15 (1–51, 10)	11 (1–45, 10)	**0.003**
≥ 10/ < 10	116/37	54/45	**0.002**
Child–Pugh score	5 (5–6, 0)	5 (5–8, 0)	0.352

Continuous data are presented as median (range, interquartile range) or mean ± standard deviation, whereas categorical data are shown as number of patients. Significant *P* values are in boldface

BMI, body mass index; ASA, American Society of Anesthesiologists; HCC, hepatocellular carcinoma; CCC, cholangiocellular carcinoma; meta, liver metastasis; HCV, hepatitis C virus; HBV, hepatitis B virus; ASH, alcoholic steatohepatitis; NASH, non-alcoholic steatohepatitis; ICGR15, indocyanine green retention rate at 15 min

^a^Data on liver fibrosis were partially lacking (monopolar, 113 cases; bipolar, 84 cases)

^b^Data on the size of the largest nodule and number of nodules were partially lacking because biliary tract cancer and benign disease could not be accurately measured (monopolar, 133 cases; bipolar, 97 cases)

^c^Data on C-reactive protein were partially lacking (monopolar, 154 cases; bipolar, 79 cases)

^d^Data on ICGR15 were partially lacking (monopolar, 153 cases; bipolar, 99 cases)

### Patients’ characteristics after PSM

Because differences in preoperative parameters have the potential to affect the postoperative course after liver resection, PSM was performed between the two groups. After matching, 73 well-balanced patients in each group demonstrated similar results (Table 2).

**Table 2:** Patient characteristics after propensity score matching

	Monopolar (n = 73)	Bipolar (n = 73)	*P* value
Age	70 (31–87, 13)	69 (38–85, 16)	0.994
Sex (male/female)	52/21	51/22	0.856
BMI	22.2 (15.4–32.2, 4.20)	22.8 (15.9–37.1, 4.50)	0.768
ASA (1/2/3)	5/56/12	9/54/10	0.506
Diagnosis (HCC/CCC/meta/biliary tract cancer/benign disease/other)	41/7/15/2/8/0	46/4/15/3/5/0	0.246
Background liver disease (normal/HCV/HBV/ASH/NASH/other)	35/22/6/3/4/3	30/28/8/2/3/2	0.475
Liver fibrosis^a^ (f0–1, f2–4)	29/33	23/38	0.309
Type of resection (partial/lateral/subsegmentectomy/segmentectomy/hemihepatectectomy/trisegmentectomy)	17/1/25/10/20/0	18/1/25/10/19/0	0.433
Number of resected lesions	1 (1–16, 0)	1 (1–3, 0)	0.365
Size of the largest nodule on preoperative imaging^b^ (cm)	2.7 (0.9–20, 3.0)	2.4 (0.5–10, 1.9)	0.147
Number of nodules on preoperative imaging^b^	1 (1–10, 0)	1 (1–10, 1)	0.513
Lymph node dissection (yes/no)	11/62	9/64	0.630
Biliary reconstruction (yes/no)	5/68	6/67	0.754
Intraoperative drain insert (yes/no)	73/0	72/1	0.500
Duration of drain placement	4 (0–131, 4)	5 (2–94, 3)	0.112
Neoadjuvant chemotherapy (yes/no/previous)	10/62/1	11/60/2	0.584
Total bilirubin (mg/dL) (≥ 1.1/ < 1.1)	16/57	17/56	0.843
Aspartate transaminase (IU/L) (≥ 31/ < 31)	36/37	38/35	0.741
Albumin (g/dL) (≥ 3.9/ < 3.9)	26/47	26/47	1.000
Prothrombin activity (%) (≥ 69/ < 69)	69/4	71/2	0.340
Activated partial thromboplastin time (%) (≥ 69/ < 69)	62/11	65/8	0.461
Hemoglobin (g/dL) (≥ 11.4/ < 11.4)	58/15	59/14	0.836
Platelet (× 10^4^/μL) (≥ 15.3/ < 15.3)	45/28	43/30	0.735
Blood urea nitrogen (mg/dL) (≥ 20.0/ < 20.0)	11/62	10/63	0.814
Creatinine (mg/dL) (≥ 1.07/ < 1.07)	14/59	9/64	0.256
C-reactive protein^c^ (mg/dL) (≥ 0.14/ < 0.14)	28/43	22/35	0.923
ICGR15^d^ (%) (≥ 10/ < 10)	45/25	45/25	0.557
Child–Pugh score	5 (5–6, 0)	5 (5–8, 0)	0.084

Continuous data are presented as median (range, interquartile range), whereas categorical data are shown as number of patients

BMI, body mass index; ASA, American Society of Anesthesiologists; HCC, hepatocellular carcinoma; CCC, cholangiocellular carcinoma; meta, liver metastasis; HCV, hepatitis C virus; HBV, hepatitis B virus; ASH, alcoholic steatohepatitis; NASH, non-alcoholic steatohepatitis; ICGR15, indocyanine green retention rate at 15 min

^a^Data on liver fibrosis were partially lacking (monopolar, 62 cases; bipolar, 61 cases)

^b^Data on the size of the largest nodule and number of nodules were partially lacking because biliary tract cancer and benign disease could not be accurately measured (monopolar, 66 cases; bipolar, 68 cases)

^c^Data on C-reactive protein were partially lacking (monopolar, 71 cases; bipolar, 57 cases)

^d^Data on ICGR15 were partially lacking (monopolar, 70 cases; bipolar, 70 cases)

### Intraoperative outcomes and postoperative blood examination

**Fig. 3:**
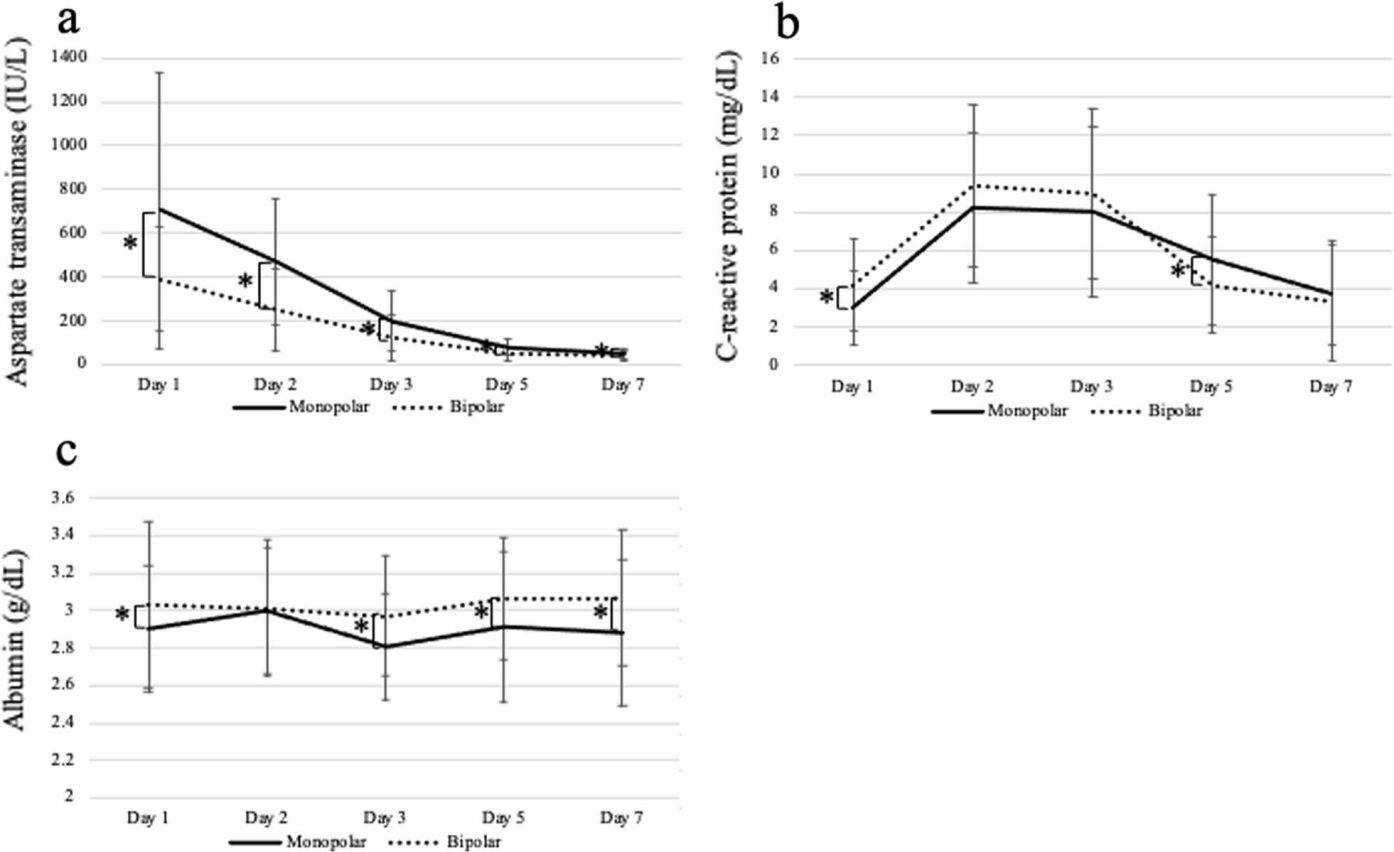
Postoperative blood examination. Postoperative blood examinations are shown as a black line for the monopolar group and a dotted black line for the bipolar group. Error bars indicate standard error. Asterisks indicate significance (*P < 0.05)

Table 3 shows the intraoperative outcomes after PSM. The total operative time (239 [74–673] vs. 275 [89–562] min; *P* = 0.013), volume of blood loss (487 [0–3275] vs. 790 [145–8030] mL; *P* < 0.001), and volume of red blood cell transfusion (0 [0–1120] vs. 0 [0–3360] mL; *P* = 0.002) were significantly higher in the bipolar group than in the monopolar group. Statistical interactions between hemostatic device and postoperative day were shown for each parameter, including AST, CRP, and albumin (Table 4). All statistical differences in AST between each postoperative day were significant (Fig. 3a, Tables 4 and 5), except from days 5 to 7. The AST level on each postoperative day was significantly higher in the monopolar group than in the bipolar group (Fig. 3a, Table 4). When focusing on postoperative day 5, the level of CRP was significantly higher in the monopolar group than in the bipolar group (Fig. 3b, Table 4). Nutritional status was significantly poorer in the monopolar group than in the bipolar group on postoperative days 1, 3, 5, and 7 (Fig. 3c, Table 4).

**Table 3:** Intraoperative outcomes

	Monopolar (n = 73)	Bipolar (n = 73)	*P* value
Total operative time (min)	239 (74–673, 130)	275 (89–562, 115)	**0.013**
Transection time^a^ (min)	76 (15–298, 61)	90 (20–240, 67)	0.104
Pringle maneuver^b^ (min)	54 (15–158, 30)	66 (18–159, 35)	0.101
Blood loss (mL)	487 (0–3275, 480)	790 (145–8030, 832)	** < 0.001**
Red blood cell transfusion (mL)	0 (0–1120, 0)	0 (0–3360, 320)	**0.002**

Continuous data are presented as median (range, interquartile range). *P* values were calculated using the Mann–Whitney *U* test. Significant *P* values are in boldface

^a^Data on total transection time were partially lacking (monopolar, 58 cases; bipolar, 67 cases)

^b^Data on total Pringle maneuver time were partially lacking (monopolar, 46 cases; bipolar, 53 cases)

**Table 4:** Linear mixed-effects model analysis between hemostatic device and postoperative day for each parameter

	Monopolar			Bipolar			*P* value^a^	*P* value^b^	*P* value^c^
Parameter	Average	SE	95% CI	Average	SE	95% CI			
Aspartate transaminase	298	21	257, 339	167	20.8	125, 208	** < 0.001**	** < 0.001**	** < 0.001**
C-reactive protein	5.8	0.30	5.1, 6.4	6.0	0.3	5.3, 6.6	0.602	** < 0.001**	** < 0.001**
Albumin	2.9	0.04	2.8, 3.0	3.0	0.04	3.0, 3.1	**0.020**	0.135	**0.026**

*P* values were calculated using the linear mixed-effects model for repeated measures. Significant *P* values are in boldface

SE, standard error; CI, confidence interval

^a^*P* value between hemostatic devices

^b^*P* value between postoperative days

^c^*P* value between hemostatic device and postoperative day

**Table 5:** Postoperative aspartate transaminase course

	Average	SE	95% CI
Day 1	546.4	21.9	503.4, 589.3
Day 2	360.1	22.4	316.0, 404.1
Day 3	158.9	27.4	105.1, 212.6
Day 5	53.1	26.3	1.4, 104.7
Day 7	42.6	22.3	− 1.1, 86.4

SE, standard error; CI, confidence interval

### Incidence and severity of postoperative morbidity

Throughout the postoperative course, the overall postoperative complication rates and the incidence rates of severe clinically relevant complications (CD grade ≥ 3) tended to be higher in the monopolar group than in the bipolar group (63.0% vs. 47.9%, *P* = 0.067, and 24.7% vs. 13.7%, *P* = 0.093, respectively; Table 6). Moreover, the prevalence of ascites was significantly higher in the monopolar group than in the bipolar group (27.4% vs. 8.2%; *P* = 0.002). Severe intra-abdominal infection occurred more frequently as a complication in the monopolar group than in the bipolar group (12.3% vs. 2.7%; *P* = 0.028). Furthermore, the severity of postoperative morbidity, as evaluated using the CCI score, was significantly higher in the monopolar group than in the bipolar group (8.7 [0.00–100] vs. 0.0 [0.00–52.4]; *P* = 0.032).

**Table 6:** Postoperative outcomes

	Monopolar (n = 73)	Bipolar (n = 73)	*P* value
Operative mortality (yes/no)	3/70	0/73	0.122
Operative morbidity (yes/no)	46/27	35/38	0.067
Grade ≥ 3 operative morbidity (yes/no)	18/55	10/63	0.093
Pleural effusion (yes/no)	8/65	15/58	0.112
Grade ≥ 3 pleural effusion (yes/no)	0/73	0/73	–
Ascites (yes/no)	20/53	6/67	**0.002**
Grade ≥ 3 ascites (yes/no)	1/72	0/73	0.500
Biliary leak (yes/no)	6/67	6/67	1.000
Grade ≥ 3 biliary leak (yes/no)	6/67	6/67	1.000
Pneumonia (yes/no)	5/68	1/72	0.104
Grade ≥ 3 pneumonia (yes/no)	2/71	0/73	0.250
Intra-abdominal infection (yes/no)	10/63	6/67	0.289
Grade ≥ 3 intra-abdominal infection (yes/no)	9/64	2/71	**0.028**
Surgical site infection (yes/no)	11/62	14/59	0.510
Grade ≥ 3 surgical site infection (yes/no)	1/72	2/71	0.500
Liver failure (yes/no)	4/69	0/73	0.060
Grade ≥ 3 liver failure (yes/no)	3/70	0/73	0.122
CCI score	8.70 (0.00–100, 26.2)	0.00 (0.00–52.4, 20.9)	**0.032**
Hospital stay (days)	16 (4–93, 18)	15 (8–99, 9)	0.370

Continuous data are presented as median (range, interquartile range), whereas categorical data are shown as number of patients. P-values were calculated using Pearson’s chi-square test or Fisher’s exact test or the Mann–Whitney *U* test when appropriate. Significant P-values are in boldface

CCI, comprehensive complication index

### Risk factors for ascites and grade ≥ 3 intra-abdominal infection

Clinical characteristics stratified by ascites are presented in Table 7. Diagnosis, background liver condition, prothrombin activity (≥ 69% or < 69%), platelet count (≥ 15.3 × 10^4^/μL or < 15.3 × 10^4^/μL), Child–Pugh score, and hemostatic device use were identified as significant risk factors for ascites in the univariate analysis. Multivariate logistic regression analysis showed that the type of hemostatic device used (OR 5.626; 95% CI 1.881–16.827; *P* = 0.002) was an independent risk factor. The association between grade ≥ 3 intra-abdominal infection and perioperative characteristics is shown in Table 8. The results indicated that lymph node dissection (OR 8.661; 95% CI 1.985–37.794; *P* = 0.004), AST level (≥ 31 or < 31 IU/L) (OR 0.151; 95% CI 0.028–0.823; *P* = 0.029), and type of hemostatic device used (OR 5.905; 95% CI 1.096–31.825; *P* = 0.039) were independent risk factors for postoperative complications.

**Table 7:** Univariate and multivariate analyses for predicting ascites

Variables	Ascites			Multivariate analysis		
	Yes (n = 26)	No (n = 120)	*P* value	OR	95% CI	*P* value
Age	70 (31–79, 13)	69 (33–87, 15)	0.276			
Sex (male/female)	19/7	84/36	0.755			
BMI	21.3 (18.3–26.9, 4.5)	22.9 (15.4–37.1, 4.5)	0.162			
ASA (1 or 2/3)	20/6	104/16	0.214			
Diagnosis (HCC/non-HCC)	22/4	65/55	**0.007**	3.442	0.751–15.768	0.111
Background liver condition (hepatitis/normal)	21/5	60/60	**0.007**	1.246	0.292–5.328	0.766
Type of resection (major hepatectomy^a^/minor hepatectomy^b^)	19/7	90/30	0.838			
Number of resected lesions	1 (1–3, 1)	1 (1–16, 0)	0.979			
Size of the largest nodule on preoperative imaging^c^ (cm)	2.3 (0.9–20.0, 2.7)	2.5 (0.5–12.0, 2.0)	0.582			
Number of nodules on preoperative imaging^c^	1 (1–10, 2)	1 (1–10, 0)	0.204			
Lymph node dissection (yes/no)	3/23	17/103	0.724			
Biliary reconstruction (yes/no)	1/25	10/110	0.444			
Neoadjuvant chemotherapy (yes/no or previous)	2/24	19/101	0.295			
Total bilirubin (mg/dL) (≥ 1.1/ < 1.1)	8/18	25/95	0.276			
Aspartate transaminase (IU/L) (≥ 31/ < 31)	16/10	58/62	0.225			
Albumin (g/dL) (≥ 3.9/ < 3.9)	13/13	81/39	0.095			
Prothrombin activity (%) (≥ 69/ < 69)	22/4	118/2	**0.008**	0.275	0.032–2.330	0.236
Activated partial thromboplastin time (%) (≥ 69/ < 69)	22/4	105/15	0.692			
Hemoglobin (g/dL) (≥ 11.4/ < 11.4)	21/5	96/24	0.929			
Platelet (× 10^4^/μL) (≥ 15.3/ < 15.3)	8/18	80/40	**0.010**	0.359	0.123–1.050	0.061
Blood urea nitrogen (mg/dL) (≥ 20.0/ < 20.0)	5/21	16/104	0.440			
Creatinine (mg/dL) (≥ 1.07/ < 1.07)	3/23	20/100	0.518			
C-reactive protein^d^ (mg/dL) (≥ 0.14/ < 0.14)	10/15	40/63	0.915			
ICGR15^e^ (%) (≥ 10/ < 10)	19/7	71/43	0.588			
Child–Pugh score	5 (5–6, 1)	5 (5–8, 0)	**0.006**	0.509	0.191–1.354	0.176
Total operative time (min)	257 (123–673, 167)	253 (74–670, 115)	0.397			
Transection time^f^ (min)	96 (19–262, 77)	84 (15–298, 63)	0.682			
Pringle maneuver^g^ (min)	54 (18–158, 51)	58 (15–159, 31)	0.962			
Blood loss (mL)	565 (35–8030, 997)	550 (0–5940, 629)	0.269			
Red blood cell transfusion (mL)	0 (0–3360, 90)	0 (0–2850, 0)	0.218			
Hemostatic device (monopolar/bipolar)	20/6	53/67	**0.004**	5.626	1.881–16.827	**0.002**

Continuous data are presented as median (range, interquartile range), whereas categorical data are shown as number of patients. Significant P-values are in boldface

BMI, body mass index; ASA, American Society of Anesthesiologists; HCC, hepatocellular carcinoma; ICGR15, indocyanine green retention rate at 15 min; OR, odds ratio; CI, confidence interval

^a^Major hepatectomy indicates more than one section hepatectomy excluding left lateral hepatectomy

^b^Minor hepatectomy indicates partial and left lateral hepatectomy

^c^Data on the size of the largest nodule and number of nodules were partially lacking because biliary tract cancer and benign disease could not be accurately measured (with ascites, 24 cases; without ascites, 110 cases)

^d^Data on C-reactive protein were partially lacking (with ascites, 25 cases; without ascites, 103 cases)

^e^Data on ICGR15 were partially lacking (with ascites, 26 cases; without ascites, 114 cases)

^f^Data on total transection time were partially lacking (with ascites, 21 cases; without ascites, 105 cases)

^g^Data on total Pringle maneuver time were partially lacking (with ascites, 19 cases; without ascites, 80 cases)

**Table 8:** Univariate and multivariate analyses for predicting grade ≥ 3 intra-abdominal infection

Variables	Grade ≥ 3 intra-abdominal infection			Multivariate analysis		
	Yes (n = 11)	No (n = 135)	*P* value	OR	95% CI	*P* value
Age	68 (37–84, 12)	70 (31–87, 15)	0.439			
Sex (male/female)	9/2	94/41	0.402			
BMI	24.7 (18.8–27.3, 7.4)	22.4 (15.4–37.1, 4.2)	0.368			
ASA (1 or 2/3)	10/1	114/21	0.570			
Diagnosis (HCC/non-HCC)	4/7	83/52	0.115			
Background liver condition (hepatitis/normal)	4/7	77/58	0.195			
Type of resection (major hepatectomy^a^/minor hepatectomy^b^)	10/1	99/36	0.226			
Number of resected lesions	1 (1–2, 0)	1 (1–16, 0)	0.494			
Size of the largest nodule on preoperative imaging^c^ (cm)	2.3 (1.2–5.0, 2.1)	2.5 (0.5–20.0, 2.1)	0.435			
Number of nodules on preoperative imaging^c^	1 (1–10, 1)	1 (1–10, 1)	0.577			
Lymph node dissection (yes/no)	5/6	15/120	**0.004**	8.661	1.985–37.794	**0.004**
Biliary reconstruction (yes/no)	2/9	9/126	0.184			
Neoadjuvant chemotherapy (yes/no or previous)	2/9	19/116	0.710			
Total bilirubin (mg/dL) (≥ 1.1/ < 1.1)	3/8	30/105	0.701			
Aspartate transaminase (IU/L) (≥ 31/ < 31)	2/9	72/63	**0.041**	0.151	0.028–0.823	**0.029**
Albumin (g/dL) (≥ 3.9/ < 3.9)	9/2	85/50	0.225			
Prothrombin activity (%) (≥ 69/ < 69)	11/0	129/6	0.999			
Activated partial thromboplastin time (%) (≥ 69/ < 69)	8/3	119/16	0.159			
Hemoglobin (g/dL) (≥ 11.4/ < 11.4)	10/1	107/28	0.369			
Platelet (× 10^4^/μL) (≥ 15.3/ < 15.3)	9/2	79/56	0.148			
Blood urea nitrogen (mg/dL) (≥ 20.0/ < 20.0)	1/10	20/115	0.607			
Creatinine (mg/dL) (≥ 1.07/ < 1.07)	2/9	21/114	0.818			
C-reactive protein^d^ (mg/dL) (≥ 0.14/ < 0.14)	2/9	48/69	0.156			
ICGR15^e^ (%) (≥ 10/ < 10)	4/6	86/44	0.213			
Child–Pugh score	5 (5–5, 0)	5 (5–8, 0)	0.998			
Total operative time (min)	318 (202–438, 167)	251 (74–673, 119)	0.244			
Transection time^f^ (min)	89 (50–223, 63)	84 (15–298, 67)	0.494			
Pringle maneuver^g^ (min)	61 (41–119, 50)	56 (15–159, 32)	0.286			
Blood loss (mL)	470 (120–1365, 425)	565 (0–8030, 676)	0.321			
Red blood cell transfusion (mL)	0 (0–0, 0)	0 (0–3360, 0)	0.996			
Hemostatic device (monopolar/bipolar)	9/2	64/71	**0.045**	5.905	1.096–31.825	**0.039**

Continuous data are presented as median (range, interquartile range), whereas categorical data are shown as number of patients. Significant P-values are in boldface

BMI, body mass index; ASA, American Society of Anesthesiologists; HCC, hepatocellular carcinoma; ICGR15, indocyanine green retention rate at 15 min; OR, odds ratio; CI, confidence interval

^a^Major hepatectomy indicates more than one section hepatectomy excluding left lateral hepatectomy

^b^Minor hepatectomy indicates partial and left lateral hepatectomy

^c^Data on the size of the largest nodule and number of nodules were partially lacking because biliary tract cancer and benign disease could not be accurately measured (with ascites, 8 cases; without ascites, 126 cases)

^d^Data on C-reactive protein were partially lacking (with ascites, 11 cases; without ascites, 117 cases)

^e^Data on ICGR15 were partially lacking (with ascites, 10 cases; without ascites, 130 cases)

^f^Data on total transection time were partially lacking (with ascites, 11 cases; without ascites, 115 cases)

^g^Data on total Pringle maneuver time were partially lacking (with ascites, 9 cases; without ascites, 90 cases)

### Thermal damage to the resected specimens

**Fig. 4:**
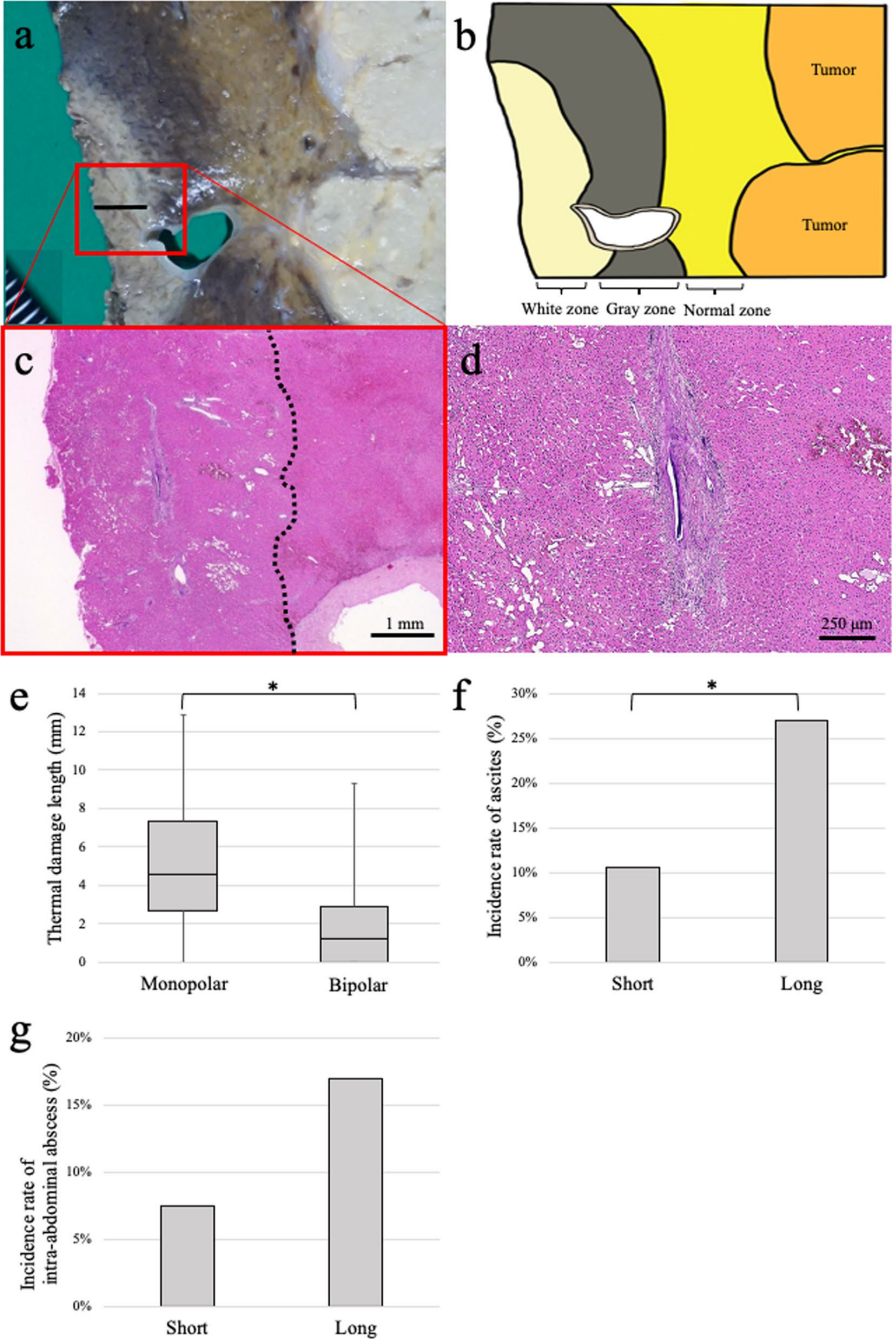
Histopathological examination of thermal damage to the resected specimens. **a** Macroscopic findings revealed that the burned area was divided into two areas from a “white zone” to a “gray zone.” The area between the “gray zone” and tumors was recognized as the “normal zone.” The measurement of the burned area is shown with the black line. **b** Illustration of macroscopic findings. **c** The black dotted line indicates the border between the “white zone” and “gray zone.” Scale bar: 1 mm. **d** Hepatocellular degeneration, dilatation of the sinusoidal space, crush degeneration of Glisson’s sheath, hemorrhage, and hyperemia were observed in the “white zone.” Scale bar: 250 μm. **e** Thermal damage length in the resected specimens caused by hemostatic devices. Horizontal lines denote median values; boxes denote the interquartile range; whiskers denote minimum and maximum values. **f** Incidence rate of ascites. **g** Incidence rate of intra-abdominal abscess. Asterisks indicate significance (*P < 0.05)

The degree of thermal damage was assessed in 146 resected specimens as an alternative measure of thermal damage to the remnant liver. Severe damage, including hepatocellular degeneration, dilatation of the sinusoidal space, crush degeneration of Glisson’s sheath, hemorrhage, and hyperemia, was detected in the white zone (Fig. 4a–d). The thermal damage observed when using a monopolar hemostatic device was significantly longer than that identified when using a bipolar hemostatic device (4.6 [0.0–13] vs. 1.2 [0.0–9.3] mm; *P* < 0.001) (Fig. 4e). Moreover, when the length of thermal damage was divided into two groups (namely, short [< 4 mm, 93 cases] and long [≥ 4 mm, 53 cases]), the incidence of ascites in the long thermal damage group was significantly higher than that in the short thermal damage group (26.4% vs. 10.8%; *P* = 0.014) (Fig. 4f). The frequency of intra-abdominal abscess also tended to be higher in the long thermal damage group than in the short thermal damage group (17.0% vs. 7.5%; *P* = 0.079) (Fig. 4g).

## Discussion

Our results indicate that the monopolar device led to higher postoperative complications than the bipolar device. This study showed that the incidence of all-grade ascites and grade ≥ 3 intra-abdominal infection was significantly higher after utilizing the monopolar device than after utilizing the bipolar device. Notably, the CCI score was significantly higher in the monopolar group than in the bipolar group.

Previous studies reported that the monopolar device was efficient and safe for decreasing surgical time and surgical bleeding without increasing complications compared with the bipolar device [12, 13]. However, caution concerning the monopolar device was advised when hyperthermia during surgery, widespread burn injury to the remnant liver, and increased postoperative transaminase level became evident. Another point of concern with the monopolar device was delayed-onset postoperative complications possibly caused by burn injury; however, no increase in major complications was observed [12]. The reason for this mismatch has not been proven.

A meta-analysis revealed that perioperative blood transfusion was associated with an elevated risk of death, recurrence, and postoperative complications in patients with hepatocellular carcinoma [22]. These findings emphasize the need for performing surgical techniques meticulously to minimize blood loss. Several methods, such as hepatic transection using the CUSA, Harmonic, LigaSure, and EnSeal sealing vessel systems, vascular occlusion via the Pringle maneuver, and intraoperative low central venous pressure, have been adopted to reduce blood loss and blood transfusion [2–4, 6–8, 10, 23–26]. By contrast, when bleeding occurs from the cut surface of the liver, a hemostatic device is needed. Several hemostatic devices, such as ones providing soft coagulation with a monopolar electrode, a monopolar floating ball (TissueLink; Salient Surgical Technologies, Inc., Portsmouth, NH, USA), a Coolinside device (Apeiron Medical, Valencia, Spain), and the bipolar forceps coagulation, have been used clinically in recent years [3, 15, 18, 27, 28]. Bipolar cautery also offers advantages in terms of reduced blood loss during hepatectomy and shortened operation time [29, 30]. Each device has its own advantage; however, to date, there is no consensus on the ideal method for hepatectomy. Therefore, hepatic surgeons select the hemostatic device according to their preference. This study aimed to clarify the advantages or disadvantages of the monopolar and bipolar coagulation devices for hemostasis during hepatectomy and postoperative complications.

We noticed several biases in terms of patient characteristics between the two groups. To minimize bias, PSM was performed according to the background liver disease, preoperative liver functions, and type of surgical procedures. This statistical procedure has been widely applied to analyze groups with different backgrounds [3, 31, 32]. The type of the energy devices was not included as a parameter in PSM and would not have affected the hepatectomy result because these devices were used only for liver mobilization and lymph node dissection, and liver parenchymal transection was performed using CUSA and manual ligation. After matching, the monopolar group showed a reduction in blood loss, transfusion volume, and total operative time. This finding indicated that the monopolar hemostatic device provided a stronger and quicker coagulative effect than the bipolar hemostatic device. On the contrary, increases in the level of AST were more frequently observed in the monopolar group than in the bipolar group. The monopolar system uses a computer-controlled low voltage level without electrical discharge, and therefore, heat is transferred to the deeper areas of the liver [12, 13]. By contrast, the bipolar system can cauterize only active bleeding between the forceps without adjacent tissue damage [16, 17]. Moreover, thermal damage to the deep cut surface of the liver can be avoided. As previously reported, when a monopolar system was continuously used during transection, hyperthermic and widespread burn injury to the remnant liver surface occurred, which can increase the postoperative transaminase levels or cause other unexpected liver dysfunctions [12, 13].

Next, we considered the individual complications associated with heat injury. One of the most critical complications after hepatectomy is an intra-abdominal infection. In the present study, a significant increase in severe intra-abdominal infection was observed in the monopolar group. However, no statistical difference in biliary leaks was detected between the two groups. Minor bile duct damage undetectable by the bile leak test may be caused by heat injury. A previous study revealed a significant increase in bile leakage with the use of a monopolar hemostatic device [33]. Another study reported a major bile duct injury caused by prolonged exposure to heat produced by the monopolar device [34]. By contrast, bipolar coagulation can prevent deep parenchymal necrosis and bile leakage induced by deep biliary necrosis [35, 36]. In a pig model, histological examination revealed that the thermal damage caused by the monopolar device was deeper than 10 mm, whereas the damage caused by the bipolar device was 2–3 mm deep [14]. In our study, the extent of thermal damage to the resected specimens was also greater in the monopolar group than in the bipolar group. Additionally, the incidence of intra-abdominal infection tended to be higher in the long thermal damage group.

Another individual complication of concern is ascites. In our study, the rate of ascites was significantly higher in the monopolar group than in the bipolar group. Damage to the remnant liver caused by monopolar devices could delay liver regeneration and prolong alleviation of inflammation, potentially leading to higher incidence rates of ascites. A previous study revealed that liver dysfunction, including low levels of serum albumin and platelet count, is a prognostic factor for the prevalence of ascites [37]. As discussed so far, extensive damage to the remnant liver can worsen liver reserve capacity and result in increase in ascites incidence.

Complications after hepatectomy are complex and are closely related to surgical manipulations, anesthesia technique, preoperative evaluation, and postoperative management [38]. In this study, multivariate analysis identified numerous clinical parameters as risk factors for ascites or severe intra-abdominal infection. Surprisingly, the type of hemostatic device used was an independent risk factor for both complications.

Finally, we assessed the overall morbidity using the CCI score, which is calculated based on the complication grading by CD classification and integrates every complication occurring after an intervention [20]. The overall morbidity is rated from 0 (no complication) to 100 (death). CD classification includes only the most serious complications; conversely, the CCI score summarizes the total postoperative complication rate associated with a surgical procedure even when multiple complications occur [39]. The CCI score is considered to be more sensitive than the CD classification when reporting postoperative morbidity in liver surgery [40]. Based on the CCI score, the severity of total complications in the monopolar group was significantly higher than that in the bipolar group. However, postoperative complications are affected by various factors other than operative procedures, energy devices, or hemostatic devices. Prospective validation study is necessary to elucidate our results.

This study has some potential limitations. First, the historical background was different; a bipolar device was used from 2009 to 2011, whereas a monopolar device was used from 2011 to 2018. The chief surgeon (TS) was the same during the entire study period, and the indications for hepatic resection and choice of procedure were decided under constant criteria. However, a learning curve in the surgical techniques and other confounders might have affected the outcomes. Second, the data were derived from a retrospective single-center cohort with a small sample size. Third, because the propensity score is a summary of measured covariates, it cannot eliminate unmeasured confounding factors. It is also difficult to completely eliminate arbitrariness by statistical adjustment. Finally, thermal damage to the resected specimens does not always correspond to damage to the remnant liver. Taking this into consideration, the results of this study should be verified by other large-scale series or multicenter randomized controlled trials. Thus, we are planning to conduct a multicenter randomized controlled trial based on this retrospective cohort study.

The results of this study may elucidate the impact of hemostatic devices and can aid surgeons in properly using surgical devices. Indeed, considering the spread of heat injury, the monopolar system should be carefully used only for pinpoint hemostasis. Furthermore, this device should not be used near the main Glissonean pedicle to prevent intra-abdominal infection or bile leakage.

## Conclusion

Although monopolar devices have an excellent hemostatic capacity, they may cause more damage to the remnant liver. The use of monopolar devices can be one of the factors that increase the frequency of complications, such as intra-abdominal infection and ascites, compared with the use of bipolar devices.

## Supplementary Information

**Additional file 1**. **Supplementary Table 1.** Classification of surgical complications for ascites. **Supplementary Table 2.** Classification of surgical complications for intra-abdominal infection.

## Data Availability

All data generated or analyzed during this study are included in this published article.

## Abbreviations

CUSA: Cavitron ultrasonic surgical aspirator
PSM: Propensity score matching
CD: Clavien-Dindo
CCI: Comprehensive complication index
SD: Standard deviation
AST: Aspartate transaminase
ICGR15: Indocyanine green retention rate at 15 min
CRP: C-reactive protein
OR: Odds ratio
CI: Confidence interval

## References

[1] Latchana N, Hircarra DH, Hallet J, Karanicolas PJ (2019). Red blood cell transfusion in liver resection. Langenbecks Arch Surg.

[2] Romano F, Franciosi C, Caprotti R, Uggeri F, Uggeri F (2005). Hepatic surgery using the Ligasure vessel sealing system. World J Surg.

[3] Hamada T, Nanashima A, Yano K, Sumida Y, Hiyoshi M, Imamura N (2017). Significance of a soft-coagulation system with monopolar electrode for hepatectomy: a retrospective two-institution study by propensity analysis. Int J Surg.

[4] Pachter HL, Spencer FC, Hofstetter SR, Coppa GF (1983). Experience with the finger fracture technique to achieve intra-hepatic hemostasis in 75 patients with severe injuries of the liver. Ann Surg.

[5] Gurusamy KS, Pamecha V, Sharma D, Davidson BR (2009). Techniques for liver parenchymal transection in liver resection. Cochrane Database Syst Rev.

[6] Doklestic K, Karamarkovic A, Stefanovic B, Stefanovic B, Milic N, Gregoric P (2012). The efficacy of three transection techniques of the liver resection: a randomized clinical trial. Hepatogastroenterology.

[7] Arru M, Pulitanò C, Aldrighetti L, Catena M, Finazzi R, Ferla G (2007). A prospective evaluation of ultrasonic dissector plus harmonic scalpel in liver resection. Am Surg.

[8] Gotohda N, Yamanaka T, Saiura A, Uesaka K, Hashimoto M, Konishi M, Shimada K (2015). Impact of energy devices during liver parenchymal transection: a multicenter randomized controlled trial. World J Surg.

[9] Hanyong S, Wanyee L, Siyuan F, Hui L, Yuan Y, Chuan L (2015). A prospective randomized controlled trial: comparison of two different methods of hepatectomy. Eur J Surg Oncol.

[10] Nanashima A, Abo T, Arai J, Takagi K, Matsumoto H, Takeshita H (2013). Usefulness of vessel-sealing devices combined with crush clamping method for hepatectomy: a retrospective cohort study. Int J Surg.

[11] Yokoo H, Miyata H, Konno H, Taketomi A, Kakisaka T, Hirahara N (2016). Models predicting the risks of six life-threatening morbidities and bile leakage in 14,970 hepatectomy patients registered in the National Clinical Database of Japan. Medicine (Baltimore).

[12] Hirokawa F, Hayashi M, Miyamoto Y, Iwamoto M, Tsunematsu I, Asakuma M (2011). A novel method using the VIO soft-coagulation system for liver resection. Surgery.

[13] Itoh S, Fukuzawa K, Shitomi Y, Okamoto M, Kinoshita T, Taketomi A (2012). Impact of the VIO system in hepatic resection for patients with hepatocellular carcinoma. Surg Today.

[14] Ikeda T, Akahoshi T, Kawanaka H, Uchiyama H, Yamashita YI, Morita M (2013). Optimum hepatic parenchymal dissection to prevent bile leak: a comparative study using electrosurgical and stapling devices in swine. Fukuoka Igaku Zasshi.

[15] Guo JY, Li DW, Liao R, Huang P, Kong XB, Wang JM (2014). Outcomes of simple saline-coupled bipolar electrocautery for hepatic resection. World J Gastroenterol.

[16] Robinson AM, Fishman AJ, Bendok BR, Richter CP (2015). Functional and physical outcomes following use of a flexible CO_2_ laser fiber and bipolar electrocautery in close proximity to the rat sciatic nerve with correlation to an in vitro thermal profile model. Biomed Res Int.

[17] Tokugawa J, Ogura K, Yatomi K, Kudo K, Hishii M, Tanikawa R (2018). Bipolar cutting method: another technique for harvesting donor artery with histological investigation. Oper Neurosurg (Hagerstown).

[18] Torzilli G, Donadon M, Marconi M, Procopio F, Palmisano A, Del Fabbro D (2008). Monopolar floating ball versus bipolar forceps for hepatic resection: a prospective randomized clinical trial. J Gastrointest Surg.

[19] Dindo D, Demartines N, Clavien PA (2004). Classification of surgical complications: a new proposal with evaluation in a cohort of 6336 patients and results of a survey. Ann Surg.

[20] Slankamenac K, Graf R, Barkun J, Puhan MA, Clavien PA (2013). The comprehensive complication index: a novel continuous scale to measure surgical morbidity. Ann Surg.

[21] Sakaguchi T, Suzuki A, Unno N, Morita Y, Oishi K, Fukumoto K (2010). Bile leak test by indocyanine green fluorescence images after hepatectomy. Am J Surg.

[22] Liu L, Wang Z, Jiang S, Shao B, Liu J, Zhang S (2013). Perioperative allogenenic blood transfusion is associated with worse clinical outcomes for hepatocellular carcinoma: a meta-analysis. PLoS ONE.

[23] Wang WD, Liang LJ, Huang XQ, Yin XY (2006). Low central venous pressure reduces blood loss in hepatectomy. World J Gastroenterol.

[24] Takayama T, Makuuchi M, Kubota K, Harihara Y, Hui AM, Sano K (2001). Randomized comparison of ultrasonic vs clamp transection of the liver. Arch Surg.

[25] Pai M, Jiao LR, Khorsandi S, Canelo R, Spalding DR, Habib NA (2008). Liver resection with bipolar radiofrequency device: Habib 4X. HPB (Oxford).

[26] Lesurtel M, Lehmann K, de Rougemont O, Clavien PA (2009). Clamping techniques and protecting strategies in liver surgery. HPB (Oxford).

[27] Ishiko T, Inomata Y, Beppu T, Asonuma K, Okajima H, Takeiti T (2012). An improved technique for liver transection using a new device for soft coagulation in living donor hepatectomy. Hepatogastroenterology.

[28] Quesada R, Poves I, Berjano E, Vilaplana C, Andaluz A, Moll X (2017). Impact of monopolar radiofrequency coagulation on intraoperative blood loss during liver resection: a prospective randomised controlled trial. Int J Hyperthermia.

[29] Rocca A, Cipriani F, Belli G, Berti S, Boggi U, Bottino V (2021). The Italian Consensus on minimally invasive simultaneous resections for synchronous liver metastasis and primary colorectal cancer: a Delphi methodology. Updates Surg.

[30] Kamarajah SK, Wilso CH, Bundred JR, Lin A, Sen G, Hammon JS (2020). A systemic review and network meta-analysis of parenchymal transection techniques during hepatectomy: an appraisal of current randomized controlled trials. HPB (Oxford).

[31] Nishizuka SS, Tamura G, Nakatochi M, Fukushima N, Ohmori Y, Sumida C (2018). Helicobacter pylori infection is associated with favorable outcome in advanced gastric cancer patients treated with S-1 adjuvant chemotherapy. J Surg Oncol.

[32] Chhatriwalla AK, Amin AP, Kennedy KF, House JA, Cohen DJ, Rao SV (2013). Association between bleeding events and in-hospital mortality after percutaneous coronary intervention. JAMA.

[33] Kaibori M, Shimizu J, Hayashi M, Nakai T, Ishizaki M, Matsui K (2015). Late-onset bile leakage after hepatic resection. Surgery.

[34] Gerlach TW, Troppmann C, Khatri VP (2009). Major bile duct injury resulting from radiofrequency-assisted hepatectomy. Hepatogastroenterology.

[35] Takahashi H, Akyuz M, Aksoy E, Aucejo F, Quintini C, Miller C (2018). A new technique for hepatic parenchymal transection using an articulating bipolar 5 cm radiofrequency device: results from the first 100 procedures. HPB (Oxford).

[36] Yamamoto T, Uenishi T, Kaneda K, Okawa M, Tanaka S, Kubo S (2017). Secure, low-cost technique for laparoscopic hepatic resection using the crush-clamp method with a bipolar sealer. Asian J Endosc Surg.

[37] Yoshikawa T, Nomi T, Hokuto D, Yasuda S, Kawaguchi C, Yamada T (2017). Risk factor for postoperative ascites in patients undergoing liver resection for hepatocellular carcinoma. World J Surg.

[38] Jin S, Fu Q, Wuyun G, Wuyun T (2013). Management of post-hepatectomy complications. World J Gastroenterol.

[39] de la Plaza LR, Ramia Ángel JM, Bellón JM, Arteaga Peralta V, García Amador C, López Marcano AJ (2018). Clinical validation of the comprehensive complication index as a measure of postoperative morbidity at a surgical department: a prospective study. Ann Surg.

[40] Giani A, Cipriani F, Famularo S, Donadon M, Bernasconi DP, Ardito F (2020). Performance of comprehensive complication index and Clavien-Dindo complication scoring system in liver surgery for hepatocellular carcinoma. Cancers (Basel).

