# Supplementary material for: Comparison of operative outcomes between monopolar and bipolar coagulation in hepatectomy: a propensity score-matched analysis in a single center
Source: BMC Gastroenterol. 2022 Mar 29;22:154. doi: 10.1186/s12876-022-02231-y (PMC8962169; doi:10.1186/s12876-022-02231-y)
Supplement: Supplementary file 1 — Additional file 1. Supplementary Table 1. Classification of surgical complications for ascites. Supplementary Table 2. Classification of surgical complications for intra-abdominal infection. [file 12876_2022_2231_MOESM1_ESM.docx]

**Supplementary Table 1. Classification of surgical complications for ascites**

| **Grade** | **Definition** |
| --- | --- |
| Grade I | Any deviation from the normal postoperative course without the need for |
|  | pharmacological treatment or surgical, endoscopic, and radiological interventions |
|  | Allowed therapeutic regimen is diuretics. |
| Grade II | Requiring pharmacological treatment with drugs other than those allowed for grade I complications |
|  | Blood transfusions and total parenteral nutrition are also included. |
| Grade III | Requiring surgical, endoscopic, or radiological interventions |
|  | The above intervention includes drain placement or puncture. |
| Grade IIIa | Intervention not under general anesthesia |
| Grade IIIb | Intervention under general anesthesia |
| Grade IV | Life-threatening complication requiring IC/ICU management |
| Grade IVa | Single-organ dysfunction |
| Grade IVb | Multiorgan dysfunction |
| Grade V | Death of the patient |

IC, intermediate care; ICU, intensive care unit

**Supplementary Table 2. Classification of surgical complications for intra-abdominal infection**

| **Grade** | **Definition** |
| --- | --- |
| Grade I | Any deviation from the normal postoperative course without the need for |
|  | pharmacological treatment or surgical, endoscopic, and radiological interventions |
|  | Allowed therapeutic regimen is antipyretics and analgesics. |
| Grade II | Requiring pharmacological treatment with drugs other than those allowed for grade I complications |
| Grade III | Requiring surgical, endoscopic, or radiological interventions |
|  | The above intervention includes drain placement or puncture. |
| Grade IIIa | Intervention not under general anesthesia |
| Grade IIIb | Intervention under general anesthesia |
| Grade IV | Life-threatening complication requiring IC/ICU management |
| Grade IVa | Single-organ dysfunction |
| Grade IVb | Multiorgan dysfunction |
| Grade V | Death of the patient |

IC, intermediate care; ICU, intensive care unit
